# *Priestia flexa* as a Novel Urinary Tract Pathogen in Daloa, Côte d'Ivoire: Insights From Genomic Sequencing

**DOI:** 10.1155/2024/6239250

**Published:** 2024-08-03

**Authors:** Dého Aristide Gbégbé, Okran Beyosse Christophe Kacou, N'goran Parfait N'zi, Djédoux Maxime Angaman

**Affiliations:** ^1^ Department of Biochemistry-Microbiology Jean Lorougnon Guédé University, Daloa, Côte d'Ivoire; ^2^ Department of Bacteriology-Virology National Reference Center for Antibiotics Institut Pasteur, Abidjan, Côte d'Ivoire

**Keywords:** antibiotic resistance, comparative genomics, Côte d'Ivoire, genomic sequencing, *Priestia flexa*, urinary tract infections, virulence factor

## Abstract

Bacterial strains coded 21LM367, 21LM07, and 21LM1136 were isolated from the urine of patients with urinary tract infections (UTIs) at the Centre Hospitalier Régional de Daloa in Côte d'Ivoire. Based on average nucleotide identity (ANI) analysis, DNA-DNA digital hybridisation (dDDH), and other comparative genomic methods, strains 21LM07, 21LM367, and 21LM1136 were determined to be *Priestia flexa*. The size of the assembled complete genomes ranged from 8,624,538 to 4,007,501 bp. The average GC content was 37.76%, 46.33%, and 43.03% for strains 21LM07, 21LM367, and 21LM1136, respectively. The total number of coding regions (CDS) in each genome was 4172, 8497, and 6795, respectively, for strains 21LM07, 21LM367, and 21LM1136. Genomic prediction analysis revealed that a total of 4241, 8583, and 6881 genes were annotated in the 21LM07, 21LM367, and 21LM1136 genomes, respectively. No virulence or resistance genes were predicted in the genomes of strains 21LM07 and 21LM1136. On the other hand, two genes conferring resistance to beta-lactam and tetracyclines as well as nine virulence genes were predicted in the genome of 21LM367. In addition, 438, 350, and 153 mobile genetic elements (MGEs) were predicted in the genomes of strains 21LM367, 21LM1136, and 21LM07, respectively. Strain 21LM07 was characterised by the absence of plasmids in its genome. Two plasmids were predicted in the genomes of isolates 21LM367 and 21LM1136; however, rep7a and IncI2 were predicted to contain the tet(K) resistance gene. No typical multilocus sequences could be characterised in the genomes of the different strains.

## 1. Introduction

Urinary tract infections (UTIs) are characterised by abnormal proliferation of micro-organisms in the urinary tract and affect any part of the urinary system, including the bladder, kidneys, urethra, or ureters [1, 2]. UTIs manifest as pain or burning on urination, increased frequency of urination, cloudy or foul-smelling urine, and sometimes fever or back pain [3]. These conditions play a major role in nephrological pathology because of their frequency in women and men of all ages. They affect around 405 million people worldwide, causing 0.23 million deaths and costing more than $6 billion in treatment and lost productivity each year, with drastic consequences such as kidney failure [4–7]. The micro-organisms that cause UTIs are varied, with significant changes from 1 year to the next and differences between countries and regions [8]. However, most UTIs are caused by bacteria, the most common of which are members of the Enterobacteriaceae family, led by uropathogenic *Escherichia coli*, which is responsible for around 80% of uncomplicated UTIs, 95% of nosocomial infections, and half of all hospital-acquired infections [9–12]. In Côte d'Ivoire, as in most developing countries, the diagnosis of UTIs is essentially based on urine cytobacteriological examination (UCT). However, interpretation of the results of the UBEC is often fraught with inconsistencies and difficulties, leading to diagnostic errors and inappropriate antibiotic prescriptions [13]. These errors lead to false positives and false negatives. False negatives result in undertreatment of UTIs, which can lead to severe sepsis and renal sequelae, while false positives lead to a lack of awareness of the true diagnosis, increased costs of managing UTIs, and exposure of patients to the side effects of antibiotics [14]. Excessive use of antibiotics for suspected UTIs has a significant impact on bacterial ecology, leading to an increase in bacterial resistance [15, 16]. However, the epidemiology and microbial ecology of UTIs in Côte d'Ivoire remain underexplored. Several factors such as local climate, healthcare practices, and antibiotic use patterns may contribute to a particular microbial environment, potentially harboring bacterial strains with distinct characteristics. Studying these local bacterial populations is crucial to developing strategies for diagnosing, treating, and preventing these conditions in the country. In addition, understanding the genetic make-up and resistance profiles of these strains can provide insight into the wider mechanisms of bacterial adaptation and antibiotic resistance. The main objective of this study is to identify and characterise new bacterial strains implicated in UTIs in Côte d'Ivoire. This research is aimed at elucidating the genetic traits of bacterial isolates implicated in UTIs, in particular their antibiotic resistance and virulence factors, using advanced molecular techniques such as next-generation sequencing (NGS) and comprehensive bioinformatics tools. NGS technologies enable a more thorough and efficient exploration of the genome, although they raise challenges in terms of access and study [17]. These advances have major implications in various fields, including medicine, biology, and genetic research, providing a more precise understanding of the genetic basis of diseases and opening the way to new therapeutic approaches. Today, genomic typing approaches based on the sequencing of complete genomes provide very high resolution, notably with core genome multilocus sequence typing (MLST), whole genome MLST, and, above all, SNP-based phylogeny. In so doing, this study is aimed at contributing to a global understanding of bacterial diversity in UTIs and the evolutionary landscape of antibiotic resistance.

## 2. Materials and Methods

### 2.1. Study Design

A comprehensive genome-wide comparison was conducted on three bacterial isolates (21LM07, 21LM1136, and 21LM367) obtained from patients with UTIs at the Daloa Regional Hospital. These isolates, collected in 2021, were identified as Gram-positive bacilli and have been preserved in the Bacteriology-Virology Laboratory at the Daloa Regional Hospital. Genomic sequencing and subsequent analysis were performed to thoroughly characterise their genomic profiles and assess their potential pathogenicity and resistance mechanisms.

### 2.2. DNA Extraction

Genomic DNA (gDNA) was extracted from bacterial samples using the cetyltrimethylammonium bromide (CTAB) method [18]. Initially, the inoculated broth, cultured for 18–24 h, was centrifuged at 10,000 rpm for 5 min to pellet the cells. The supernatant was discarded, and the pellet resuspended in 1.5 mL of CTAB lysis buffer (CTAB1; pH 8.0) containing 20 g/L CTAB, 1.4 M NaCl, 0.1 M Tris, and 0.02 M Na-EDTA. Subsequently, 5 *μ*L of 20 mg/mL RNase was added, and the mixture was homogenized using a vortex mixer. The homogenate was incubated at 60°C (±2) for 30 min, with intermittent shaking after 15 min to ensure resuspension. After this initial incubation, 10 *μ*L of 20 mg/mL proteinase K was added and the mixture was vortexed again. A second incubation at 60°C (±2) for another 30 min included stirring after 15 min to resuspend the contents. Postincubation, the homogenate was centrifuged at 15,000 g for 10 min. A 900 *μ*L aliquot of the supernatant was transferred to a new tube, mixed with an equal volume of chloroform, and vortexed. After centrifugation at 15,000 g for 15 min, 650 *μ*L of the clear supernatant was transferred to a new tube and mixed with 1300 *μ*L of precipitation buffer (CTAB2; 5 g/L CTAB and 0.04 M NaCl). The mixture was incubated at room temperature for 1 h and centrifuged at 15,000 g for 15 min. The supernatant was discarded, and the pellet was resuspended in CTAB3 buffer (NaCl) and 700 *μ*L of chloroform. This was homogenized for 30 s and centrifuged at 15,000 g for 10 min. Subsequently, 600 *μ*L of the aqueous phase was mixed with 360 *μ*L of cold isopropanol (−20°C) and inverted several times. After a 20-min incubation at room temperature, the sample was centrifuged at 15,000 g for 15 min to pellet the DNA. The supernatant was removed, and the DNA pellet was washed with 500 *μ*L of 70% ethanol, gently inverting the tube several times before another centrifugation at 15,000 g for 10 min. The supernatant was discarded, and the DNA was dried in an oven at 55°C for 30 min to evaporate residual ethanol. The DNA was then rehydrated to a concentration of 50 ng/*μ*L in PCR-grade water for subsequent sequencing analysis.

### 2.3. Genome Sequencing and Assembly

The bacterial genomes were sequenced using Illumina's NextSeq platform. DNA libraries for NGS were prepared using the ZymoBIOMICS DNA Kit from Zymo Research. Quality of extracted DNA was assessed with a NanoDrop One9+ spectrophotometer. The gDNA samples underwent fragmentation via an enzymatic method provided in the NEBNext® Ultra™ II FS DNA Library Preparation Kit for Illumina systems. Resulting DNA fragments were purified using AMPure XP beads and underwent end-repair processes. Subsequently, Illumina-specific adaptor sequences were ligated to the fragments. Postligation, the DNA fragments were quantified and individually indexed, followed by a secondary size-selection step using AMPure XP beads to ensure library uniformity. Library quality was verified on an Agilent TapeStation system using a DNA chip. Sequencing was performed on the NextSeq platform utilizing a NextSeq 300-cycle kit. Postsequencing, the raw reads were subjected to quality control, including adapter trimming and filtering, using the Fastp tool (Version 0.23.4 + galaxy0) within the Galaxy Europe framework [19]. De novo assembly of the filtered raw reads was conducted using the Unicycler tool (Galaxy Version 0.5.0 + galaxy1), with all parameters set to default [20]. The quality and integrity of the assembled genomes were evaluated using QUAST (Galaxy Version 5.2.0 + galaxy1) to ensure accurate assembly metrics [21, 22].

### 2.4. Taxonomic Identification of Bacterial Isolates and Phylogenetic Analysis

To ensure the reliability of the genomic data, the CheckM tool (Galaxy Version 1.2.0 + galaxy0) was employed to assess genome completeness and contamination, using default parameters [23]. This initial evaluation helped establish the quality of the genomes for subsequent analyses. For determining the taxonomic affiliations of the bacterial isolates, both DNA-DNA digital hybridisation (dDDH) and average nucleotide identity (ANI) were calculated. These metrics were derived from comparing the complete genome sequences of the study isolates to those of related type strains using the Type (Strain) Genome Server (TYGS) and JSpeciesWS (a web server for prokaryotic species circumscription based on pairwise genome comparison) online platforms [24, 25]. It is generally accepted that isolates belonging to the same species exhibit a dDDH value of at least 70% and an ANI value of at least 95% [26], serving as benchmarks for species delineation in our study. Further comparative genomics was conducted using Kraken2, which aligns fragmented nucleotides of the whole genomes against a database of reference genomes [27]. This analysis provides insights into the genetic relatedness and potential evolutionary histories of the isolates. Finally, a phylogenetic tree was constructed using MEGA 11 software, integrating multiple sequence alignments to visually represent the evolutionary relationships among the isolates based on their genomic sequences. This phylogenetic analysis not only contextualizes the taxonomic positioning of the isolates but also illustrates their evolutionary lineage within a broader microbial landscape.

### 2.5. Genome Annotation and Prediction of Resistance Genes and Virulence Factor Genes

The genomes of the bacterial isolates were annotated to identify and map gene locations and functions. This was initially facilitated by the Proksee tool [28], which generated a comprehensive genomic map for each isolate. Subsequent detailed annotations were performed using Prokka (Galaxy Version 1.14.6 + galaxy1) [29]. Prokka systematically annotates genomic elements such as coding sequences, ribosomal RNA (rRNA), and transfer RNA (tRNA) genes, providing a functional overview of the genetic architecture of the isolates.

The detection and characterisation of antibiotic resistance genes were performed using the Abricate tool, which queries the ResFinder database (Galaxy Version 1.0.1) with default settings [30]. This analysis is crucial for understanding the potential resistance mechanisms embedded within the bacterial genomes, facilitating insights into the therapeutic challenges posed by these isolates.

To identify virulence factor genes, the genomes were analyzed using the VFDB database [31]. This process involves the prediction and validation of genes associated with pathogenicity, which are crucial for assessing the virulence potential of the bacterial isolates. Only predicted genes with a similarity percentage between 97% and 100% were considered confirmed, ensuring high confidence in the identification of virulence factors.

### 2.6. Prediction of Mobile Genetic Elements (MGEs)

The identification of plasmids within the genomes of the bacterial isolates was conducted using PlasmidSPAdes [32]. Following this, plasmid contigs were selectively filtered to isolate relevant sequences. These sequences were then annotated with Prokka to detail specific genetic features and potential functions.

To ascertain the presence of antibiotic resistance genes and virulence factors within the plasmid sequences, the Abricate tool was employed, utilizing the ResFinder and VFDB databases for comprehensive gene prediction. This step is crucial for understanding the accessory genetic content that could contribute to the pathogenicity and resistance profiles of the isolates.

Alien Hunter [33] was utilized to predict regions within the genomes that may have been acquired through horizontal gene transfer. This analysis helps in identifying genetic segments that contribute to rapid evolutionary changes and adaptation in bacterial genomes.

The presence of prophage regions within the genome sequences was detected using the PHAge Search Tool Enhanced Release (PHASTER) database [34]. Identifying these regions is essential for understanding the lysogenic potential of the bacterial isolates and their role in horizontal gene transfer.

The broader scope of MGEs, beyond plasmids and prophages, was explored using mobileOG-db [35]. This database provided insights into various other types of mobile elements that could influence genome plasticity and the evolutionary capabilities of the isolates.

### 2.7. MLST

Molecular typing of strains using MLST is a standardised and discriminating technique, applied in epidemiological or phylogenetic studies. It is used to characterise the genetic relationships between bacterial strains. This technique makes it possible to compare the sequences of genes coding for metabolic enzymes in order to establish a clonal relationship between different strains. The MLST techniques developed are based on the analysis, by nucleotide sequencing, of the polymorphism of seven household genes conserved throughout the evolution of bacterial strains. By aligning the sequences at a given locus, it is possible to identify alleles that differ from one another as a result of mutations and/or recombination for each bacterial strain. The combination of alleles obtained from each selected locus is used to define a standard sequence (ST), representing a multilocus genotype. MLST was carried out by uploading the FASTA files to the pMLST tool. Household genes and type sequences were characterised using the pMLST tools [36].

## 3. Results

### 3.1. Quantification of DNA Extracts

After extraction of the gDNA the various extracts were quantified using NanoDrop. The purity of DNA extracts from isolates 21LM1136, 21LM367, 21LM07 to 260/280 was estimated at 1.94, 1.72, and 1.79 with respective concentrations of 285.10 ng/*μ*L and 173.60 ng/*μ*L.

### 3.2. Assembly Statistics

Table 1 provides a detailed overview of the genome assembly statistics for the three bacterial isolates examined in this study. An analysis conducted using QUAST revealed variability in the GC content among the isolates, ranging from 37.7% to 46.3%. The total size of the sequenced genomes also showed significant variation, extending from 4.0 to 8.6 Mb. Notably, the genome of isolate 21LM367 was approximately twice the size of that of isolate 21LM07. Furthermore, when assessing genome assembly quality metrics such as the N50, isolate 21LM367 demonstrated the highest value, indicating a more contiguous assembly compared to the other isolates.

### 3.3. Taxonomic Assignment of Bacterial Isolates and Phylogenetic Analysis

**Figure 1:**
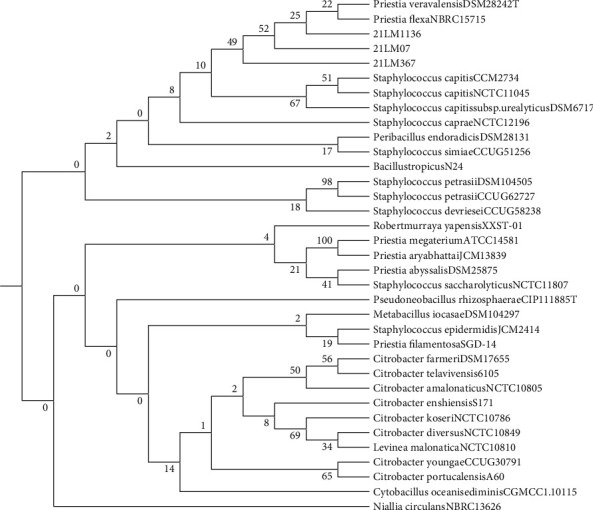
Phylogenetic tree of bacterial isolates showing the relationships between the bacterial isolates and related species, generated using MEGA 11 software.

The analysis of genome completeness and contamination, conducted using the CheckM tool, indicated high-quality assemblies with minimal heterogeneity. The genome of isolate 21LM07 showed no contamination and had a completeness of 99.43%. Isolates 21LM367 and 21LM1136 achieved completeness levels of 100% and 98.25%, respectively. However, the contamination metrics revealed that while 21LM367 had no detectable contaminants, 21LM1136 exhibited a contamination level of 76.67%. Taxonomic classification and phylogenetic relationships were established using comprehensive bioinformatics analyses. The three isolates—21LM07, 21LM367, and 21LM1136—were identified as members of the Bacillaceae family. Phylogenetic analysis of their complete genomes positioned these isolates within a clade closely related to *Priestia veravalensis* DSM28242T and *Priestia flexa* NBRC 15715, with similarity rates exceeding 98%. The GC content of the isolates varied from 37.7% to 46.3 mol%. Further differentiation using dDDH and ANI indicated high genomic similarity among the isolates. Specifically, dDDH values were around 99%, while ANI values were 97.28%, 97.89%, and 98.98% for isolates 21LM367, 21LM1136, and 21LM07, respectively, confirming them as strains of *Priestia flexa*. Comparison of whole genomes with reference genomes via the Kraken2 tool, based on the alignment of fragmented nucleotides, reinforced these findings. Fragmentation percentages for the isolates were 65.01% for 21LM367, 56.60% for 21LM1136, and 84.06% for 21LM07, further substantiating their classification as *Priestia flexa* strains. The resulting phylogenetic tree, illustrating the evolutionary relationships among these isolates and related species, is depicted in Figure 1.

### 3.4. Characteristics of Different Genomes

The comprehensive genome analysis conducted using the Prokka tool provided detailed annotations and revealed distinct genomic characteristics for each of the bacterial isolates.

The genome of isolate 21LM367 was found to be highly complex, containing a total of 8583 genes, of which 8497 were protein-coding sequences (CDS). Additionally, this isolate's genome includes 2 transfer-messenger RNAs (tmRNAs), 74 tRNAs, and 10 rRNAs. Two repeated regions were also identified, highlighting a significant level of genomic organisation and potential regulatory complexities.

Annotation of the genome of isolate 21LM1136 revealed 6881 genes, with 6795 coding for proteins. This genome also harbors 1 tmRNA, 79 tRNAs, and 6 rRNAs, suggesting a slightly different transcriptional machinery compared to 21LM367, which may influence its physiological and pathogenic capabilities.

**Figure 2:**
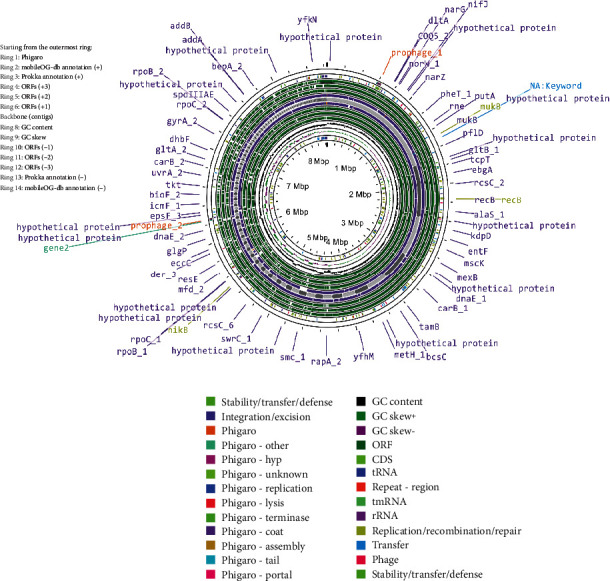
Genomic visualization of *Priestia flexa* isolate 21LM367, annotated using Proksee, highlighting annotations and mobile genetic elements.

**Figure 3:**
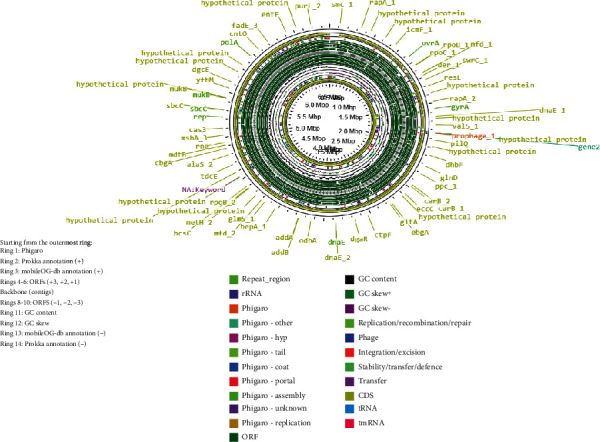
Genomic visualization of *Priestia flexa* isolate 21LM1136, annotated using Proksee, highlighting annotations and mobile genetic elements.

**Figure 4:**
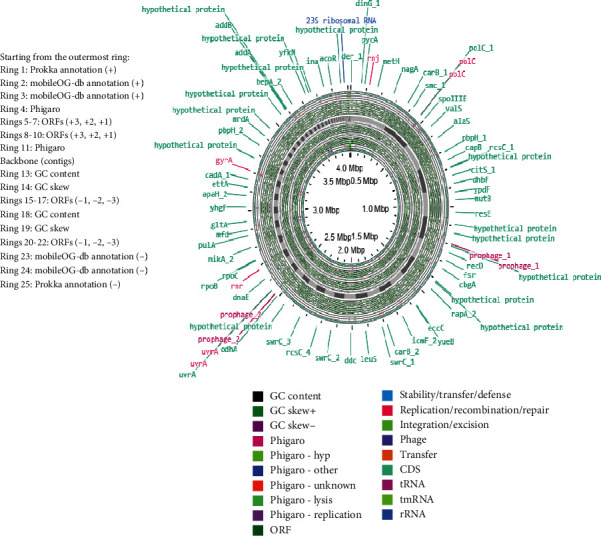
Genomic visualization of *Priestia flexa* isolate 21LM07, annotated using Proksee, highlighting annotations and mobile genetic elements.

The smallest genome among the three, that of isolate 21LM07, comprises 4241 genes with 4172 protein-coding sequences. It contains 1 tmRNA, 66 tRNAs, and only 2 rRNAs, which might reflect its streamlined genomic structure tailored to specific environmental or host-related adaptations. The annotated genomes are shown in Figures 2, 3, and 4.

### 3.5. Prediction of Resistance and Virulence Genes of Bacterial Isolates

This analysis focused on the prediction of antibiotic resistance and virulence genes within the genomes of three *Priestia flexa* strains, based on data retrieved from the ResFinder and VFDB databases. The criteria for inclusion required that the predicted genes exhibit a similarity percentage between 97% and 100%.

For strains coded 21LM1136 and 21LM07, no resistance or virulence genes were identified in the respective databases. In contrast, the genome of strain 21LM367 exhibited a notable presence of resistance genes. Specifically, two genes were identified:

− blaMAL-1_2 gene: Conferring resistance to beta-lactam antibiotics, this gene was found with a 100% coverage and 98.34% identity, registered under the accession number AJ609506.
− tet(K) gene: Associated with resistance to tetracycline, this gene also showed a 100% coverage and identity, cataloged under the accession number U38656.

Additionally, nine virulence factors were predicted in the genome of 21LM367, prominently including yersiniabactin. These findings are detailed in Table 2 and highlight the potential pathogenic capabilities of this particular strain.

### 3.6. Prediction of MGEs and Characterisation of Resistance Genes and Virulence Factors Linked to These Elements

The characterisation of MGEs in the genomes of the three bacterial isolates was extensively analyzed and is summarized in Table 3.

The plasmidFinder tool was employed to detect plasmids within the genomes of isolates 21LM367 and 21LM1136, predicting the presence of IncI2 and rep7a plasmid sequences. In contrast, isolate 21LM07 was notable for the absence of any plasmid sequences.

Analysis using ResFinder identified the tet(K) gene, which confers resistance to tetracycline, in isolates 21LM367 and 21LM1136. This gene demonstrated 100% identity and coverage and was cataloged under the accession number U38656.

Unique among the isolates, 21LM1136 harbored the SenB gene on its plasmids, identified as an enterotoxin with 99.91% coverage and 98.59% identity, indicating a significant virulence potential linked to MGEs.

Analysis predicted significant horizontal gene transfer regions across the genomes, with isolate 21LM367 exhibiting 244 regions, isolate 21LM1136 with 202 regions, and isolate 21LM07 with 136 regions, highlighting the dynamic genomic architecture and potential for genetic exchange.

The genomic sequences of the isolates revealed the presence of prophage regions. Isolate 21LM367 contained one intact prophage region similar to PHAGE-Entero-mEp237-NC-019704. Isolates 21LM07 and 21LM1136 both exhibited one intact and one incomplete prophage region each, with the intact regions resembling PHAGE-Bacill-PM1-NC-020883 and the incomplete ones similar to PHAGE-Brevib-Sundance-NC-028749 and PHAGE-Geobac-E3-NC-029073, respectively.

A total of 438, 350, and 153 MGEs were predicted in the genomes of isolates 21LM367, 21LM1136, and 21LM07, respectively, illustrating a significant variation in the presence and type of mobile genetic content across the isolates.

### 3.7. MLST

The results of MLST in the genomes of the three isolates submitted for this study using the pMLST tool revealed the absence of housekeeping genes in isolate 21LM07. MLST was also unable to reveal the type of sequence. MLST typing of the genome of isolates 21LM1136 and 21LM367 predicted the presence of seven housekeeping genes, namely, aspC(252), clpX(~281), fadD(293), mdh(256), arcA(107), dnaG(~230), and lysP(271).

## 4. Discussion

UTIs represent a crucial public health problem because of their drastic impact on the renal health of individuals [37]. Accurate identification of the microorganisms involved in these conditions in developing countries such as Côte d'Ivoire is therefore essential in order to guide the competent authorities towards effective patient management methods. Despite the usefulness of routine phenotypic or molecular typing methods, NGS offers extraordinary insight into the genomic organisation of the pathogens that cause epidemics. The data generated by genome sequencing can be used to track clinically important pathogens circulating in the population in the town of Daloa. However, studies based on these cutting-edge techniques and exploring the dynamic organisation of bacterial genomes are virtually nonexistent in Côte d'Ivoire. Therefore, the aim of this study was to characterise the strains involved in UTIs using their whole genome and to assess the distribution of their virulence gene, antibiotic resistance gene, and MGEs. On the basis of ANI analysis, dDDH, and other comparative genomics methods, it was determined that the strains 21LM07, 21LM367, and 21LM1136 submitted for study were *Priestia flexa*. This bacterium, *Priestia flexa*, is a Gram-positive halophytic bacillus and mangrove endophyte [38]. Prized for its biotechnological applications such as the development of bioplastics, the production of bioethanol, bioremediation of chromium-contaminated environments, and the amelioration of arsenic-related stress in the growth of *Oryza sativa* L, no study has yet revealed the involvement of *Priestia flexa* in UTIs [38–41]. However, recent research has isolated *Priestia flexa* from human faeces, suggesting that the human intestine could be a reservoir for this bacterium [42]. This discovery could explain its presence in UTIs in Daloa, through a potential transfer from the anal region to the bladder. Following analysis of the different genomes, variation in genetic content within the genomes was observed. Prediction analysis showed a variation in GC content of between 37.7% and 46.3% and in genome size of between 4 and 8.6 Mb. This variability in genome size and GC content within the same species could be due to contamination of the sequences of isolates 21LM1136 and 21LM367 or to the increased presence of xenologous genes in these organisms. Annotation of the different genomes predicted that the genome of coded bacterial isolate 21LM367 contained 8583 genes and 8497 coding regions (CDS), two (2) repeat regions, 2 tmRNAs, 74 tRNAs, and 10 rRNAs. The genome of isolate 21LM1136 contained 6881 genes and 6795 CDS, 1 tmRNA, 79 tRNA, and 6 rRNA and two repeat regions. In contrast, the genome of isolate 21LM07 contained 4241 genes, 4172 CDS, 1 tmRNA, 66 tRNA, and 2 rRNA. The genomic characteristics of the *Priestia flexa* strains observed in this study are very different from those observed in the *Priestia* sp. strain isolated in Mexico [43]. The presence of different RNAs, the most abundant of which were tRNAs, shows the pathogenicity potential of the different bacterial isolates studied, as the development of an infection essentially depends on the coordinated and sequential expression of a multitude of virulence factors and accessory genes. This coordination is ensured by an extremely complex regulatory network made up of protein factors as well as regulatory RNAs [44]. The presence of repeated sequences in the genomes of isolates 21LM367 and 21LM1136 is thought to be due to their large size. The study revealed that a variation in the presence of antibiotic resistance genes was found between the genomes compared. No resistance or virulence genes were observed in the genome of strain 21LM07. Resistance was acquired in strain 21LM1136. However, the combined action of intrinsic and acquired resistance was observed in strain 21LM367. This combination of intrinsic and acquired resistance observed in isolate 21LM367 would be a serious obstacle to the treatment of infections caused by this bacterial strain. In addition, the blaMAL-1_2 and tet(K) genes conferred intrinsic resistance in strain 21LM367 to beta-lactams and tetracyclines. Overall, the acquired resistance observed in strains 21LM367 and 21LM1136 was mediated by the rep7a and IncI2 plasmids and characterised by the tet(K) gene. The pathogenicity of strain 21LM367 was conferred by 9 genes. The pathogenicity of this strain is thought to result from the concerted action of several virulence factors. Elsewhere in the world, data on the epidemiology of *Priestia flexa* resistance to antibiotics is scarce, given its low involvement in infections. Nevertheless, a recent study showed that a *Priestia flexa* isolate called *Priestia flexa* KS1 was resistant to cefixime, clavulanic acid/ceftazidime, nafalline, methicillin, trimethoprim, kanamycin, and nalidix antibiotics and degraded mucin under in vitro conditions and acclimatised better in the gastrointestinal environment [42]. However, the genes conferring resistance in *Priestia flexa* KS1 and their location were not elucidated by this study. Analysis of bacterial MGEs in genomic sequences is a critical step towards profiling the underlying causes of antibiotic resistance, phenotypic or metabolic diversity, and the evolution of bacterial genera. The prediction of MGEs in the sequences of the three strains in the study resulted in the recording of a total of 438, 350, and 153 MGEs in the sequences of 21LM367, 21LM1136, and 21LM1136, respectively. The results of this study show the presence of integron cassettes in association with islands of resistance, highlighting the dynamic nature of the genomes. Among these MGEs, the presence of phage regions was reported. In the genome of isolate 21LM367, only one intact phage region was predicted, and this region was similar to PHAGE-Entero-mEp237-NC-019704 (16). On the other hand, in the genomes of isolates 21LM07 and 21LM1136, the presence of one intact phage region and one incomplete phage region was demonstrated. The predicted intact phage regions were similar to PHAGE-Bacill-PM1-NC-020883 (12). The incomplete phage regions were similar to PHAGE-Brevib-Sundance-NC-028749(2) and PHAGE-Geobac-E3-NC-029073(2), respectively. The role of bacterial prophages in the transfer of resistance genes has been reported in several studies [45]. However, in this study, no resistance genes were predicted in these prophage regions. Nevertheless, further study is needed to analyze the potential of these prophage regions in the transfer of resistance genes and virulence in these strains. In the present study, the presence of plasmids, in particular the rep7a and IncI2 plasmids, was reported in the genomes of isolates 21LM1136 and 21LM367, associated with the putative tet(K) gene conferring resistance to doxycycline and tetracycline. The SenB (enterotoxin) gene was also predicted as a virulence factor linked to plasmids from isolate 21LM1136. This presence of plasmid-mediated resistance and virulence genes would be due to horizontal gene transfer. In order to establish the clonal relationships between the different strains of *Priestia flexa* involved in UTIs in the town of Daloa, the nucleotide sequence variation of seven housekeeping genes was determined. The MLST typing results predicted the presence of seven housekeeping genes, aspC(252), clpX(~281), fadD(293), mdh(256), arcA(107), dnaG(~230), and lysP(271), in the genomes of isolates 21LM1136 and 21LM367 and an absence of a housekeeping gene in the genome of isolate 21LM07, suggesting that isolate 21LM367 is related to isolate 21LM1136.

## 5. Conclusion

This study conclusively identified *Priestia flexa* in the urine of patients from the Centre Hospitalier Régional de Daloa, marking the first documented instance of this bacterium as a pathogen in UTIs in both Daloa and the broader Côte d'Ivoire region. This discovery underscores the critical role of advanced genomic sequencing techniques in uncovering the diversity of infectious agents involved in UTIs, enhancing our understanding of their epidemiology. The identification of *Priestia flexa* not only broadens the known spectrum of UTI pathogens but also provides a crucial foundation for further epidemiological studies in Daloa and across Côte d'Ivoire. The findings emphasize the necessity for ongoing surveillance and research to improve clinical management and outcomes of UTIs, highlighting the dynamic and complex nature of bacterial infections within the urinary tract.

## Figures and Tables

**Table 1:** Statistics of assembled genomes providing a detailed overview of the genome assembly metrics for the bacterial isolates 21LM07, 21LM1136, and 21LM367, including the number of contigs, total length, N50 values, and GC content.

**Isolates**	**Metrics**	**Values**
21LM07	# contigs	99
	# contigs (≥ 0 bp)	156
	# contigs (≥ 1000 bp)	91
	Largest contig	298,311
	Total length (bp)	4,007,501
	Total length (≥ 0 bp)	4,019,830
	Total length (≥ 1000 bp)	4,002,254
	N50	96,329
	GC (%)	37.76

21LM1136	# contigs	440
	# contigs (≥ 0 bp)	449
	# contigs (≥ 1000 bp)	435
	Largest contig	151,904
	Total length (bp)	6,712,893
	Total length (≥ 0 bp)	6,714,677
	Total length (≥ 1000 bp)	6,709,337
	N50	27,220
	GC (%)	44.03

21LM367	# contigs	212
	# contigs (≥ 0 bp)	279
	# contigs (≥ 1000 bp)	194
	Largest contig	1,700,642
	Total length (bp)	8,624,538
	Total length (≥ 0 bp)	8,638,832
	Total length (≥ 1000 bp)	8,612,201
	N50	129,253
	GC (%)	46.33

**Table 2:** Virulence genes identified in the genome of *Priestia flexa* isolate 21LM367, including gene names, percentage identity, accession numbers, and the associated products.

**Gene**	**%identity**	**Accession**	**Product**
*fyuA*	98.57	NP_405467	(fyuA) pesticin/yersiniabactin receptor protein (yersiniabactin [VF0136]) (*Yersinia pestis* CO92)
*ybtE*	97.84	NP_405468	(ybtE) yersiniabactin siderophore biosynthetic protein (yersiniabactin [VF0136]) (*Yersinia pestis* CO92)
*ybtU*	99.36	NP_405470	(ybtU) yersiniabactin biosynthetic protein YbtU (yersiniabactin [VF0136]) (*Yersinia pestis* CO92)
*irp1*	98.59	NP_405471	(irp1) yersiniabactin biosynthetic protein Irp1 (yersiniabactin [VF0136]) (*Yersinia pestis* CO92)
*irp2*	98.84	NP_405472	(irp2) yersiniabactin biosynthetic protein Irp2 (yersiniabactin [VF0136]) (*Yersinia pestis* CO92)
*ybtA*	99.27	NP_405473	(ybtA) transcriptional regulator YbtA (yersiniabactin [VF0136]) (*Yersinia pestis* CO92)
*ybtP*	98.67	NP_405474	(ybtP) lipoprotein inner membrane ABC-transporter (yersiniabactin [VF0136]) (*Yersinia pestis* CO92)
*ybtQ*	98.22	NP_405475	(ybtQ) inner membrane ABC-transporter YbtQ (yersiniabactin [VF0136]) (*Yersinia pestis* CO92)
*ybtX*	98.36	NP_405476	(ybtX) putative signal transducer (yersiniabactin [VF0136]) (*Yersinia pestis* CO92)

**Table 3:** Mobile genetic elements found in the genomes of the bacterial isolates 21LM07, 21LM1136, and 21LM367, categorized by function such as integration/excision, replication/recombination/repair, phage, stability/transfer/defense, and transfer.

**Isolates**	**Mobile genetic elements**	**Number**
21LM07	Integration/excision	55
	Replication/recombination/repair	46
	Phage	25
	Stability/transfer/defense	08
	Transfer	19
	Plasmides	00

21LM1136	Integration/excision	55
	Replication/recombination/repair	133
	Phage	55
	Stability/transfer/defense	31
	Transfer	76
	Plasmides	02

21LM367	Integration/excision	76
	Replication/recombination/repair	163
	Phage	68
	Stability/transfer/defense	39
	Transfer	92
	Plasmides	02

## Data Availability

Genomic sequence data are deposited in NCBI Sequence Read Archive under the BioProject PRJNA1069903 and BioSample accession SAMN41213920.

## References

[1] Kalinderi K., Delkos D., Kalinderis M., Athanasiadis A., Kalogiannidis I. (2018). Urinary tract infection during pregnancy: current concepts on a common multifaceted problem. *Journal of Obstetrics and Gynaecology*.

[2] Bargotya M., Kumar L., Kachhap P., Das P., Sachdeva V., Bhattar S. A. (2020). A flow cytometric and cytochemistric analysis of urine to detect early urinary tract infection. *Annals of Pathology and Laboratory Medicine*.

[3] Foxman B. (2002). Epidemiology of urinary tract infections. Incidence, morbidity, and economic costs. *The American Journal of Medicine*.

[4] Wagenlehner F., Tandogdu Z., Bartoletti R. (2016). The global prevalence of infections in urology study: a long-term, worldwide surveillance study on urological infections. *Pathogens*.

[5] Belete M. A., Saravanan M. (2020). A systematic review on drug resistant urinary tract infection among pregnant women in developing countries in Africa and Asia; 2005-2016. *Infection and Drug Resistance*.

[6] Islam M. A., Islam M. R., Khan R. (2022). Prevalence, etiology and antibiotic resistance patterns of community-acquired urinary tract infections in Dhaka, Bangladesh. *PLoS One*.

[7] Chemlal A., Ismaili F. A., Karimi I. (2015). Les infections urinaires chez les patients insuffisants rénaux chroniques hospitalisés au service de néphrologie: profil bactériologique et facteurs de risque. *Pan African Medical Journal*.

[8] Behzadi P., Behzadi E., Ranjbar R. (2015). Urinary tract infections and *Candida albicans*. *Central European Journal of Urology*.

[9] Flores-Mireles A. L., Walker J. N., Caparon M., Hultgren S. J. (2015). Urinary tract infections: epidemiology, mechanisms of infection and treatment options. *Nature Reviews Microbiology*.

[10] Kucheria R., Dasgupta P., Sacks S., Khan M., Sheerin N. (2005). Urinary tract infections: new insights into a common problem. *Postgraduate Medical Journal*.

[11] Dhakal B. K., Kulesus R. R., Mulvey M. A. (2008). Mechanisms and consequences of bladder cell invasion by uropathogenic *Escherichia coli*. *European Journal of Clinical Investigation*.

[12] Foxman B. (2010). The epidemiology of urinary tract infection. *Nature Reviews Urology*.

[13] Leroy V., Mariani-Kurkdjian P. (2004). Epidemiology and diagnosis of urinary tract infections. *Médecine Thérapeutique Pédiatrie*.

[14] Lammers R. L., Gibson S., Kovacs D., Sears W., Strachan G. (2001). Comparison of test characteristics of urine dipstick and urinalysis at various test cutoff points. *Annals of Emergency Medicine*.

[15] Fenwick E. A. L., Briggs A. H., Hawke C. I. (2000). Management of urinary tract infection in general practice: a cost-effectiveness analysis. *British Journal of General Practice*.

[16] Freeman J. T., Anderson D. J., Sexton D. J. (2009). Seasonal peaks in *Escherichia coli* infections: possible explanations and implications. *Clinical Microbiology and Infection*.

[17] Mardis E. R. (2008). The impact of next-generation sequencing technology on genetics. *Trends in Genetics*.

[18] Huang X., Duan N., Xu H., Xie T. N., Xue Y.-R., Liu C.-H. (2018). DNA extraction from fungi with high polysaccharide content using CTAB-PEG. *Molecular Biology*.

[19] Chen S., Zhou Y., Chen Y., Gu J. (2018). Fastp: an ultra-fast all-in-one FASTQ pre-processor. *Bioinformatics*.

[20] Wick R. R., Judd L. M., Gorrie C. L., Holt K. E. (2017). Unicycler: resolving bacterial genome assemblies from short and long sequencing reads. *PLoS Computational Biology*.

[21] Mikheenko A., Prjibelski A., Saveliev V., Antipov D., Gurevich A. (2018). Versatile genome assembly evaluation with QUASTLG. *Bioinformatics*.

[22] Gurevich A., Saveliev V., Vyahhi N., Tesler G. (2013). QUAST: quality assessment tool for genome assemblies. *Bioinformatics*.

[23] Parks D. H., Imelfort M., Skennerton C. T., Hugenholtz P., Tyson G. W. (2015). CheckM: assessing the quality of microbial genomes recovered from isolates, single cells, and metagenomes. *Genome Research*.

[24] Meier-Kolthoff J. P., Goker M. (2019). TYGS is an automated high-throughput platform for state-of-the-art genome-based taxonomy. *Nature Communications*.

[25] Richter M., Rossello-Mora R., Oliver Glockner F., Peplies J. (2016). JSpeciesWS: a web server for prokaryotic species circumscription based on pairwise genome comparison. *Bioinformatics*.

[26] Orata F. D., Meier-Kolthoff J. P., Sauvageau D., Stein L. Y. (2018). Phylogenomic analysis of the Gammaproteobacterial methanotrophs (order Methylococcales) calls for the reclassification of members at the genus and species levels. *Frontiers in Microbiology*.

[27] Wood D. E., Salzberg S. L. (2014). Kraken: ultrafast metagenomic sequence classification using exact alignments. *Genome Biology*.

[28] Grant J. R., Enns E., Marinier E. (2023). Proksee: in-depth characterization and visualization of bacterial genomes. *Nucleic Acids Research*.

[29] Seemann T. (2014). Genome analysis Prokka: rapid prokaryotic genome annotation. *Bioinformatics*.

[30] Bortolaia V., Kaas R. S., Ruppe E. (2020). ResFinder 4.0 for predictions of phenotypes from genotypes. *Journal of Antimicrobial Chemotherapy*.

[31] Liu B., Zheng D., Jin Q., Chen L., Yang J. (2019). VFDB 2019: a comparative pathogenomic platform with an interactive web interface. *Nucleic Acids Research*.

[32] Antipov D., Hartwick N., Shen M., Raiko M., Lapidus A., Pevzner P. A. (2016). PlasmidSPAdes: assembling plasmids from whole genome sequencing data. *Bioinformatics*.

[33] Vernikos G. S., Parkhill J. (2006). Interpolated variable order motifs for identification of horizontally acquired DNA: revisiting the Salmonella pathogenicity islands. *Bioinformatics*.

[34] Arndt D., Marcu A., Liang Y., Wishart D. S. (2019). PHAST, PHASTER and PHASTEST: tools for finding prophage in bacterial genomes. *Briefings in Bioinformatics*.

[35] Brown C. L., Mullet J., Hindi F. (2022). mobileOG-db: a manually curated database of protein families mediating the life cycle of bacterial mobile genetic elements. *Applied and Environmental Microbiology*.

[36] Carattoli A., Zankari E., Garcia-Fernandez A. (2014). *In silico* detection and typing of plasmids using plasmidfinder and plasmid multilocus sequence typing. *Antimicrobial Agents and Chemotherapy*.

[37] Kouassi-M'bengue A., Folquet-Amorissani M., Nassirou F. (2008). Neonatal urinary tract infections in Abidjan: the problem of bacterial resistance. *Mali Médical*.

[38] Chathalingath N., Kingsly J. S., Gunasekar A. (2023). Biosynthesis and biodegradation of poly(3-hydroxybutyrate) from *Priestia flexa*: A promising mangrove halophyte towards the development of sustainable eco-friendly bioplastics. *Microbiological Research*.

[39] Shukla A., Gupta A., Srivastava S. (2023). A bacterial consortium (*Priestia endophytica* NDAS01F, *Bacillus licheniformis* NDSA24R, and *Priestia flexa* NDAS28R) and thiourea improved arsenic stress tolerance and enhanced the growth of Oryza sativa L.. *Plant Physiology and Biochemistry*.

[40] UbiIcône D. S., Icône M. G. E., Ikharia E. J., Akwagiobe E. A., Asitok A. D., Antai S. P. (2024). Production, characterization, and bio-ethanologenic potential of a novel tripartite raw starch-digesting amylase from *Priestia flexa* UCCM 00132. *Preparative Biochemistry & Biotechnology*.

[41] Ravishankar P., Srinivas Ravi M., Bharathi K., Subramanian S. K., Asiedu S. K., Selvaraj D. (2024). Unlocking nature's toolbox: kinetin-producing *Priestia flexa* VL1 paves the way for efficient bioremediation of chromium-contaminated environments. *Journal of Environmental Chemical Engineering*.

[42] Deswal G., Selwal M. K., Nirvan H., Selwal K. K. (2023). *Priestia flexa* KS1: a new bacterial strain isolated from human faeces implicated in mucin degradation. *International Microbiology*.

[43] Ortega-Urquieta M. E., Valenzuela-Ruiz V., Mitra D. (2022). Draft genome sequence of *Priestia sp*. strain TSO9, a plant growth-promoting bacterium associated with wheat (*Triticum turgidum* subsp. *durum*) in the Yaqui Valley, Mexico. *Plants*.

[44] Lays C., Romilly C., Tomasini A. (2014). L’ARN non codant RsaA favorise la persistance et atténue la virulence de *Staphylococcus aureus*. *Médecine Sciences*.

[45] Haaber J., Leisner J. J., Cohn M. T. (2016). Bacterial viruses enable their host to acquire antibiotic resistance genes from neighbouring cells. *Nature Communications*.

